# Modulating Heart Rate Variability through Deep Breathing Exercises and Transcutaneous Auricular Vagus Nerve Stimulation: A Study in Healthy Participants and in Patients with Rheumatoid Arthritis or Systemic Lupus Erythematosus

**DOI:** 10.3390/s22207884

**Published:** 2022-10-17

**Authors:** Mette Kjeldsgaard Jensen, Sally Søgaard Andersen, Stine Søgaard Andersen, Caroline Hundborg Liboriussen, Salome Kristensen, Mads Jochumsen

**Affiliations:** 1 Department of Health Science and Technology, Aalborg University, 9220 Aalborg, Denmark; 2 Department of Rheumatology, Aalborg University Hospital, 9000 Aalborg, Denmark

**Keywords:** rheumatoid arthritis, systemic lupus erythematosus, deep breathing, heart-rate variability, vagus nerve stimulation, neuromodulation, transcutaneous auricular stimulation, breathing, inflammation, autoimmune diseases

## Abstract

Rheumatoid arthritis (RA) and systemic lupus erythematosus (SLE) are associated with an impaired autonomic nervous system and vagus nerve function. Electrical or physiological (deep breathing—DB) vagus nerve stimulation (VNS) could be a potential treatment approach, but no direct comparison has been made. In this study, the effect of transcutaneous auricular VNS (taVNS) and DB on vagal tone was compared in healthy participants and RA or SLE patients. The vagal tone was estimated using time-domain heart-rate variability (HRV) parameters. Forty-two healthy participants and 52 patients performed 30 min of DB and 30 min of taVNS on separate days. HRV was recorded before and immediately after each intervention. For the healthy participants, all HRV parameters increased after DB (SDNN + RMSSD: 21–46%), while one HRV parameter increased after taVNS (SDNN: 16%). For the patients, all HRV parameters increased after both DB (17–31%) and taVNS (18–25%), with no differences between the two types of VNS. DB was associated with the largest elevation of the HRV parameters in healthy participants, while both types of VNS led to elevated HRV parameters in the patients. The findings support a potential use of VNS as a new treatment approach, but the clinical effects need to be investigated in future studies.

## 1. Introduction

Rheumatoid arthritis (RA) and systemic lupus erythematosus (SLE) are autoimmune inflammatory diseases. Patients with RA suffer from joint swelling, joint pain, and, over time, joint destruction [1]. Patients with SLE have numerous symptoms from different organ systems, including symptoms from the kidneys, joints, blood vessels, and the central nervous system [2]. These diseases can be severe and result in great suffering among patients, and studies indicate that these patients have a decreased quality of life [3,4]. Furthermore, these diseases entail a considerable societal burden [4,5].

The usual treatment options for both diseases include disease-modifying anti-rheumatic drugs (DMARD), glucocorticoids, and immunotherapy [2,6]. However, 20–25% of patients with RA and approximately 15% of patients with SLE cannot achieve remission [6,7], and these drugs can have severe side effects, such as increased rate of infection, hepatotoxicity, and bone marrow depression [8]. Consequently, it is evident that there is a need for additional treatment options.

Over the last few years, a new approach in the form of vagus nerve stimulation (VNS) has been investigated [9,10]. VNS is believed to exhibit an anti-inflammatory effect via the inflammatory reflex; afferent vagal fibers sense peripheral cytokines, which activate three pathways: (1) activation of the hypothalamus–pituitary–adrenal axis, which results in release of cortisol; (2) activation of efferent vagal fibers, which act on enteric neurons that, via activation of nicotinergic receptors on macrophages, inhibit the release of proinflammatory cytokines (e.g., Tumor Necrosis Factor Alpha (TNF-α)); (3) activation of vagal efferent fibers that stimulate the splenic nerve in the celiac ganglion. The splenic nerve stimulates β2 adrenergic receptors on splenic lymphocytes, which, in turn, inhibit macrophage release of proinflammatory cytokines in the spleen via activation of nicotinergic acetylcholine receptors [11]. Since VNS is believed to exert an anti-inflammatory effect, VNS could potentially reduce disease activity in autoimmune diseases such as RA and SLE. Another aspect that supports this notion, is the autonomic imbalance related to these diseases. Studies indicate that the autonomic nervous system is out of balance in patients with RA and SLE with a decrease in vagal tone [12]. Koopman et al. even found evidence suggesting that autonomic nervous system imbalance precedes and predicts the RA diagnosis [13].

The activity of the autonomic nervous system can be estimated non-invasively by measuring heart-rate variability (HRV). HRV is the physiological occurrence of slightly varying distances between adjacent R-waves in an electrocardiogram (ECG) recording; the time-domain measures of RMSSD and PNN50 are related to vagal activity and may be considered as surrogate measures for the vagal tone [14].

That which was mentioned above implies that patients with RA and SLE could benefit from VNS. Electrical VNS can be delivered either invasively through a cuff electrode implanted around the nerve or non-invasively via transcutaneous stimulation at auricular or cervical points. For transcutaneous auricular VNS (taVNS) in healthy subjects, the results are mixed; some studies have found an increase in different HRV measures [15,16,17,18], while other studies, including a meta-analysis, found no effect on HRV measures [19,20,21,22]. Aside from investigating the effect of taVNS on HRV, Sclocco et al. also examined the effect of taVNS on brainstem MRI in healthy subjects and found activation of the ipsilateral nucleus tractus solitarius [22]. This effect is believed to arise through activation of afferent vagal fibers at the site of stimulation in the ear [23], and it could possibly evoke a more generalized activation of the vagus nerve (VN), potentially resulting in an anti-inflammatory response, as well as an increase in HRV, thereby providing a physiological background for the effect of taVNS. For transcutaneous cervical VNS (tcVNS), two studies have reported a reduction of cytokine levels in healthy participants [24,25]. Kox et al. studied transvenous VNS in healthy participants and found no effect on the inflammatory response to endotoxemia [26]. Electrical VNS has been investigated in RA or SLE in a few studies. Koopman et al. implanted a cervical vagus nerve stimulator in 17 patients with RA and showed that 42 days of stimulation decreased cytokine levels, C-reactive protein (CRP), and Disease Activity Score-28-CRP (DAS28-CRP) [27]. Aranow et al. studied four days of taVNS in patients with SLE and showed a reduction in fatigue, pain, and number of affected joints compared to sham stimulation [28]. A study including patients with RA found that four days of tcVNS reduced cytokine levels and decreased DAS28-CRP [29]. In summary, studies investigating electrical VNS in healthy subjects report mixed results, but studies regarding electrical VNS in patients with RA or SLE have shown promising results on clinical outcomes.

Another way to stimulate the VN is through deep breathing (DB) exercises [30,31,32,33,34,35,36,37]. The mechanisms through which DB stimulates the VN are not well established, but are believed to include peripheral factors, such as the baroreflex, the peripheral chemoreflex, the Bainbridge reflex, and the Heuring–Breuer reflex, as well as such central elements as the respiratory and cardiovascular centers in the brainstem [38]. Most of the studies that have investigated DB tested the effect on HRV, and even though the methods of DB exercises used in these studies vary distinctively, a general increase in HRV parameters was found. Only two studies investigated the effect of DB in patients with RA and SLE, where DB increased the HRV parameters [36,37]. Furthermore, Twal et al. found that yogic DB can decrease cytokine levels in saliva in healthy participants [32]. Tactile VNS through an oscillatory device placed in the cymba conchae was able to significantly reduce the amount of TNF-α in healthy participants, in addition to reducing DAS28 and CRP in patients with RA [39]. The existing evidence regarding the anti-inflammatory properties of the VN has been reviewed in several articles, including clinical studies with VNS performed in different patient populations that included patients with RA and SLE [9,10,11,40,41,42,43,44].

In summary, the existing literature generally supports the hypothesis that VNS could be a potential new treatment option for patients with autoimmune inflammatory diseases such as RA and SLE, and that there are different methods for stimulating the VN, where taVNS and DB are the most extensively researched. However, these non-invasive methods of VNS have only been studied to a limited extent in patients with RA and SLE, and the effects have not been directly compared. Therefore, the aim of this study was to compare the effect of taVNS and DB on the vagal tone measured by using HRV in patients with RA and SLE. Moreover, the effects of the two methods were compared with those in a sample of healthy participants. It should be noted that the aim of this study was not to make a direct comparison between healthy participants and patients with RA and SLE, which would require an age- and gender-matched control group for the patients. The contributions of this paper are: (1) an evaluation of the effects of VNS on HRV through DB and taVNS in healthy participants and patients with RA and SLE and (2) a direct comparison between taVNS and DB.

## 2. Materials and Methods

### 2.1. Participants

Healthy participants of ages between 18 and 85 years were eligible for inclusion. The exclusion criteria were the following: a chronic illness that demanded regular prescription-only systemic medication, heart arrhythmias, lung diseases, being unable to provide informed consent, having a history of psychiatric diseases or severe mental illness that was believed to affect the ability to participate in the study, addiction, prior addiction to opioids or euphoriants, and pregnancy.

Patients with a confirmed diagnosis of either RA or SLE who attended the Department of Rheumatology, Aalborg University Hospital, Denmark, were recruited and tested for eligibility. The inclusion criteria regarding the patients were age between 18 and 85 years, diagnosis with RA according to the American College of Rheumatology (1987 or 2010) or European League Against Rheumatism (2010) classification criteria, or diagnosis with SLE according to the American College of Rheumatology classification criteria for SLE or the Systemic Lupus International Collaborating Clinics Classification criteria for SLE. Patients could have other chronic diseases that required prescription-only systemic medication; otherwise, the exclusion criteria were the same as those described for the healthy participants.

A sample size calculation showed that 42 participants were required. This calculation was based on a significance level of 0.05, a statistical power of 0.8, and an effect size estimated from a comparable study in patients with RA or SLE [36]. Forty-four healthy volunteers and 53 patients with RA or SLE were included in this study to account for drop-outs. Two healthy participants were later excluded due to the discovery of systemic medication use. Additionally, one patient with RA was excluded because of an unknown heart arrhythmia discovered during the ECG recording. The DB and taVNS were well tolerated by all participants, and the study was completed without any adverse events. Data concerning the study participants are illustrated in Table 1. There was an age difference of 30 years between the healthy participants and the patients and a difference in the gender ratio, but the two groups were not compared. The gender ratio of the patients reflected the prevalence of these diseases, which are more prevalent in women [1,2].

### 2.2. Experimental Setup

**Figure 1:**
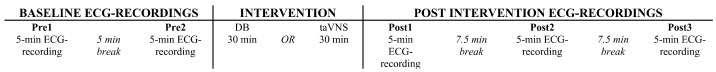
Schematization of the study design illustrating the time points of the ECG measurements, breaks, and interventions (deep breathing (DB) or transcutaneous auricular vagus nerve stimulation (taVNS)). Post1, Post2, and Post3 were measured 0–5, 12.5–17.5, and 25–30 min after the intervention, respectively.

This study was a cross-over study where both healthy volunteers and patients underwent the same experimental setup. The setup consisted of two different sessions separated by at least 24 h to avoid carry-over effects. One session involved 30 min of DB, and the other session involved 30 min of taVNS. The order of the interventions was randomized. Two ECG recordings with durations of 5 min were made prior to the intervention to establish a baseline HRV. Three ECG recordings with durations of 5 min were performed after the intervention to determine whether an immediate effect on HRV was present and whether this effect was still present up to 30 min after the intervention had ended. The study design is schematized in Figure 1. Data regarding disease activity and treatment were collected from the patients. The ECG recordings and interventions were performed in the same seating position.

### 2.3. Deep Breathing

The DB exercises were based on a procedure described in previous studies [33,36,37,45], and they consisted of 4 s of inspiration and 6 s of expiration with a total duration of 30 min. Initially, participants received verbal instructions, and during the exercise, they were guided by a visual cue. The participants were instructed to fill the lungs completely during inspiration and empty the lungs completely during expiration. The participants could freely choose between nasal or oral respiration.

### 2.4. Transcutaneous Auricular Vagus Nerve Stimulation

A NEMOS^®^ stimulator (Cerbomed, Erlangen, Germany) was used for taVNS. This is a handheld battery-driven stimulator that is placed in the cymba concha in the outer left ear, targeting the auricular branch of the vagal nerve. The device stimulates with a series of small electrical impulses with a pulse width of 250 µs and a frequency of 25 Hz for 30 min in a cycle of 30 s “on” and 30 s “off” to avoid habituation. A set stimulation of 0.5 mA was used [19]. Participants were allowed to read a book or a magazine during the taVNS.

### 2.5. Heart-Rate Variability

#### 2.5.1. ECG Recording

Based on the HRV guidelines [46], 5-min ECG recordings were used to derive the HRV. Before the first recording, the participants rested for at least 10 min. Three ECG electrodes (Ambu WhiteSensor 0415M, Ambu A/S, Ballerup, Denmark) were placed on the thorax—one on the right midclavicular line, one on the left midclavicular line, and a ground electrode medially to these. The participants were instructed to sit quietly, relax, and breathe normally during the ECG recordings. A bipolar derivation of the ECG leads was sampled with 250 Hz (OpenBCI amplifier, New York City, NY, USA) and stored on a computer for offline analysis.

#### 2.5.2. Parameter Extraction

Initially, the ECG signals were converted from the data format of the amplifier into a MATLAB (MathWorks, version 2021a, Natick, MA, USA) variable on which the remaining analyses were conducted. The 5-min recordings were bandpass filtered between 10 and 30 Hz using an 8th-order Butterworth filter to maximize the discriminability of the R-waves. The filtered ECG was loaded in the MATLAB toolbox “HRVtool” [47], where the R-waves were detected automatically. All of the identified R-waves were inspected manually to ensure that no artifacts were registered as R-waves, which would lead to an inaccurate estimate of the HRV. Incorrectly identified peaks were removed from the analysis. The following HRV parameters were extracted from HRVtool based on the R-waves: SDNN, RMSSD, and PNN50. The SDNN is the standard deviation of NN-intervals (R-R), and it reflects both sympathetic and parasympathetic activity, but with a primary source of variability through respiratory sinus arrhythmia [48]. The RMSSD is the root-mean-square of the successive difference in adjacent NN-intervals, and it was used to estimate vagally mediated changes [48]. The PNN50 is the proportion of absolute differences between successive intervals that are larger than 50 milliseconds, and it also reflects vagally mediated changes [48]. The RMSSD and PNN50 are highly correlated [46], but both measures were included in the analyses to allow for comparison with the existing literature.

### 2.6. Statistical Analysis

Initially, the data were tested for a normal distribution using a Shapiro–Wilks test, and if normality was violated, a log10 transformation (SDNN and RMSSD) or square-root transformation (PNN50, as some values were zero) was applied. A paired-sample t-test or a Wilcoxon signed rank test was performed to investigate whether a significant difference between the two baseline ECG recordings was present. If this was not the case, a mean baseline was used for further statistical analyses. To investigate the effect of DB and taVNS, one-way repeated-measure analysis of variance (rmANOVA) tests were performed, with time as a within-subject factor (four levels: baseline, Post1, Post2, and Post3) and HRV as the dependent variable. The tests were followed up with least-significant-difference post hoc analyses to reduce the type 2 error at the expense of a higher type 1 error. This was done for the two interventions, for the three HRV parameters, and in both the patient and healthy participant group, i.e., there were 12 rmANOVA tests in total. If the assumption of sphericity was violated, the Greenhouse–Geisser correction was applied. The percentage differences between the measurements before and after intervention were calculated. The percentage changes in PNN50 were considered as misleading because of the removal of subjects with the value of 0 as a baseline and because division with very small numbers would lead to very high percentage increases for some subjects, ultimately skewing the graphical results. Therefore, the percentage increase in PNN50 is not graphically displayed in the results section. These percentage differences were compared using a Wilcoxon signed rank test to investigate the difference between the effects of DB and taVNS. The comparison was made for the measurement point (Post1, Post2, or Post3) with the highest percentage increase for each intervention. In all tests, statistical significance was assumed when *p* < 0.05. The statistical analyses were performed using SPSS Statistics version 27.0 (IBM Corp., Armonk, NY, USA).

## 3. Results

### 3.1. Results: Healthy Participants

The paired-sample *t*-tests revealed no significant differences between the two pre-measurements, with *p*-values ranging from 0.502 to 0.985; thus, a mean baseline was calculated and used for further analyses.

#### 3.1.1. Effects of DB and taVNS

**Figure 2:**
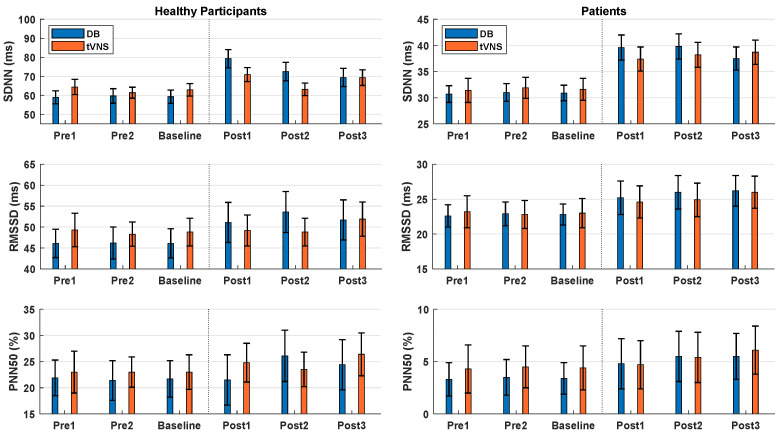
HRV data for healthy participants (**left column**) and patients (**right column**). The baseline shows the mean values of Pre1 and Pre2, which were obtained prior to the interventions. Post1, Post2, and Post3 were obtained 0–5, 12.5–17.5, and 25–30 min after the interventions, respectively. Data are displayed as the mean ± standard error across participants. DB: Deep breathing. tVNS: Transcutaneous auricular vagus nerve stimulation.

The results of the effects of DB and taVNS on HRV are summarized in Figure 2 (left column) and Table 2. The results from the rmANOVA showed a significant effect of DB regarding the HRV parameters of SDNN, RMSSD, and PNN50, which increased with respect to the baseline recordings. TaVNS caused a significant increase in SDNN, but there was generally no increase in RMSSD and PNN50—except for Post3—compared to baseline measurement. Based on Figure 2, there was no general trend of decreasing HRV values from Post1 to Post3 (indicating that the effects outlasted the stimulation period), which was also supported by the statistical analyses in Table 2. The only exception was the SDNN after DB, which decreased significantly from Post1 to Post3, although Post3 was still significantly increased compared to the baseline.

#### 3.1.2. Effect of DB Compared to taVNS

**Figure 3:**
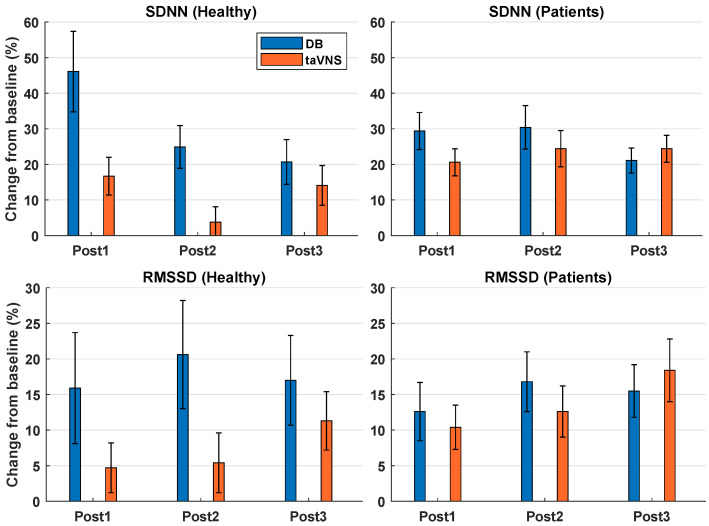
Percentage change from baseline to post-measurements for healthy participants and patients performing deep breathing (DB) or transcutaneous auricular vagus nerve stimulation (taVNS). Data are displayed as the mean ± standard error across the participants.

To compare the effects of the two interventions, the relative change between the baseline and post-measurements were calculated, and the maximal effect of each intervention was compared. The percentage changes are illustrated in Figure 3. The maximum effect of DB on the HRV parameters was found at Post1 for SDNN and at Post2 for RMSSD and PNN50. TaVNS caused a maximum effect on the HRV parameters at Post1 for SDNN and Post3 for RMSSD and PNN50. The statistical analysis revealed a significant difference (Z = −2.469, *p* = 0.014) between the maximum effects of DB (mean increase 46%) and taVNS (mean increase 16%) for SDNN (see Figure 3). No differences between the maximum effects of DB and taVNS were observed for RMSSD (Z = −0.531, *p* = 0.595) or PNN50 (Z = −0.628, *p* = 0.530). To be able to calculate and compare the percentage increase in PNN50, three subjects were not included due to the baseline measurement having a value of zero.

### 3.2. Results: Patients with RA and SLE

The paired-sample *t*-tests and Wilcoxon signed rank tests revealed no significant differences between the two pre-measurements, with *p*-values ranging from 0.216 to 0.750; thus, a mean baseline was calculated and used for further analyses.

#### 3.2.1. Effects of DB and taVNS

The results of the effects of DB and taVNS on HRV are summarized in Figure 2 (right column) and Table 3. The results from the rmANOVA showed a significant effect of DB and taVNS regarding all three HRV parameters, which increased with respect to the baseline recordings. Based on Figure 2, there was no general trend of decreasing HRV values from Post1 to Post3 (indicating that the effects outlasted the stimulation period), which was also supported by the statistical analyses in Table 3.

#### 3.2.2. Effect of DB Compared to taVNS

To compare the effects of the two interventions, the relative change between the baseline and post-measurements was calculated, and the maximal effect of each intervention was compared. The percentage changes are illustrated in Figure 3. The maximum effects of DB on SDNN, RMSSD, and PNN50 were found at Post2. Regarding taVNS, the maximum effect on SDNN was found at Post2, whereas the maximum effects on RMSSD and PNN50 were found at Post3. The statistical analyses revealed no significant differences between the maximum effects of DB and taVNS for SDNN (Z = −1.102, *p* = 0.270), RMSSD (Z = −0.674, *p* = 0.500), or PNN50 (Z = −0.639, *p* = 0.523). To be able to calculate and compare the percentage increase for PNN50, 17 subjects were not included due to the baseline measurement having a value of zero.

## 4. Discussion

In the healthy participants, DB caused a significant increase in all HRV parameters, which also corresponded to a significantly higher percentage increase in SDNN compared to taVNS. After taVNS, only SDNN was significantly increased, and only just after the intervention (Post1). SDNN reflects both sympathetic and parasympathetic activity, but depends on the length of the recording [48]; thus, it is difficult to draw specific conclusions about the vagal tone using only SDNN. Otherwise, taVNS did not cause a significant change in the HRV parameters in healthy participants according to the rmANOVA, although the pairwise comparison revealed a significant increase in RMSSD and PNN50 at Post3 compared to the baseline. Considering the above-mentioned uncertainties, our findings agree with a meta-analysis from 2021, which found no overall effect of taVNS on HRV in healthy populations compared to sham conditions [21]. This could be explained by the generally high HRV of healthy individuals, and the previous literature shows that taVNS induces a greater increase in HRV in individuals with a lower baseline HRV [49]. Moreover, the healthy participants in this study were young and, therefore, generally had a high HRV [50]. It should be noted that mixed results have been reported regarding the effect of transcutaneous VNS on HRV parameters, with both increases, decreases, and no changes (see [51] for a recent review of the literature in healthy participants and patients with various diseases). Other studies have found evidence of an anti-inflammatory effect of tcVNS in healthy participants [24,25]. Although these studies investigated tcVNS and not taVNS, this could indicate that transcutaneous VNS can cause a more generalized activation of the VN, even though the effect on cardiac vagal fibers remains uncertain.

In the patient population, both interventions increased the HRV parameters significantly, and the effects of taVNS and DB were not significantly different. In line with our results, it has previously been shown that one session of 30 min of DB significantly increased HRV parameters in patients with RA and SLE [36,37]. In the current study, the measurements were continued for up to 30 min after intervention, and at this time point, the HRV parameters were still significantly increased compared to the baseline. Interestingly, the maximum effects on HRV parameters were found at different time points for DB and taVNS. The effect of DB generally peaked at the Post2 measurement, whereas the effect of taVNS generally peaked at the Post3 measurement. This suggests that the HRV parameters could continue to rise after the post-measurements were made. This is in line with a study by Brock et al., who found a significant effect of tcVNS on cardiac vagal tone 90 min and 24 h after stimulation compared to the baseline in healthy participants [24].

It seems that both methods can stimulate the VN, and given that this activation is more generalized and not confined to the cardiac vagal fibers, DB and taVNS could potentially have an anti-inflammatory effect [10,23]. This notion is supported by the recent literature on transcutaneous VNS, where Drewes et al. found a reduction in pro-inflammatory cytokines and DAS28 after four days of tcVNS in patients with RA [29], and Aranow et al. found a reduction in pain and joint swelling in patients with SLE after four consecutive days of taVNS [28]. Addorisio et al. stimulated the VN in patients with RA with a vibrotactile device in the ear and showed a reduction in cytokine production and disease activity [39]. To our knowledge, there is a very limited number of clinical studies regarding the effect of DB on parameters such as cytokines and clinical disease activity in patients with RA or SLE. Therefore, more studies are needed to show if DB has an anti-inflammatory effect. Aside from RA and SLE, tcVNS has been investigated in other inflammatory rheumatic diseases, such as psoriatic arthritis [52] and polymyalgia rheumatica [53], providing promising results regarding clinical and biochemical outcome measures.

This study used a period of 30 min of DB, which is a relatively long period of time that demands high concentration and good compliance from the participants. Bhagat O.L. et al. found an effect after only 5 min of DB in healthy participants, but only regarding SDNN [30], whereas Sharpe et al. found an increase in RMSSD after 10 min of DB (also in healthy participants) [34]. This could indicate that an effect can be achieved in less than 30 min. This was also supported in a recent study, but it was shown that a larger effect was obtained when increasing the duration of DB from 5 to 30 min [37]. Previous studies on taVNS mainly used a stimulation period shorter than 30 min. A stimulation period of 10 min only resulted in a very limited effect on HRV parameters [20]; however, 15 min revealed a significant improvement in HRV [17,49], which indicates that a shorter stimulation period than 30 min could be used when working with taVNS in the future. Interestingly, tcVNS is generally only applied in cycles of 2 min, which has shown to still affect the cytokine level and cardiac vagal tone [24,25], thereby implying that taVNS potentially could affect clinical outcomes in a shorter stimulation period as well. This was also supported by a taVNS study using a 5-min stimulation period in patients with SLE [28].

The stimulation intensity could have been adjusted to each participant’s sensory threshold [16,49], unlike in this study, where a set stimulation of 0.5 mA was used, which meant that 13 of the patients and one healthy participant could not feel the taVNS. It has been reported that stimulation intensities below the sensory perception threshold can increase the HRV, at least for people with a low vagal modulation [51]. Nevertheless, a significant effect was found, and this was in agreement with Borges et al., who found that an individually set stimulation did not increase HRV compared to a set stimulation, and that this only increased the discomfort of the participants [19].

Our taVNS device stimulated the cymba conchae, whereas other studies have chosen to stimulate the tragus. Peuker et al. investigated the innervation of the auricle in cadavers and found that the cymba conchae was 100% vagally innervated compared to the tragus, which was only 45% vagally innervated [54]. In addition, Machetanz et al. also found that taVNS in the cymba conchae provides the best effect on HRV compared with other locations in the auricle [15], suggesting that the cymba conchae is the proper site for taVNS.

### 4.1. Limitations

It should be noted that the patients in this study generally had a low disease activity (see Table 1), and the results could be different in a population with a higher disease activity. People with a low vagal tone tend to respond better to VNS [49]; therefore, it is possible that patients with a higher disease activity would exhibit an even greater increase in HRV parameters after DB and taVNS. This study used HRV as a biomarker for vagal activity, which is, although not perfect, widely accepted and considered the best-researched biomarker so far. We included the time-domain parameters SDNN, RMSSD, and PNN50, where RMSSD and PNN50 are especially believed to express parasympathetic—and, hence, vagal—activity [23]. Other HRV measures could be included as well, such as the high-frequency component in a frequency-domain analysis, which corresponds to parasympathetic activity [23], or PNNx with an interval smaller than 50 milliseconds, e.g., 20 milliseconds, which has been shown to be good for discriminating between different conditions (heart failure/healthy heart, sleeping/awake, young/old adults) [55]. However, it should be noted that there are generally high correlations between the various HRV parameters.

As mentioned, the effect of taVNS could last longer than 30 min after the intervention and, perhaps, longer than the 24-h washout period chosen in this study [24]. However, since the order of interventions was randomized, this effect was presumably diminished. Since the durations of the effects of DB and taVNS in this study were unknown, we recorded three post measurements to pinpoint if and when the neuromodulatory effect decreased within the first 30 min after the intervention ended. This, however, caused multiple comparisons that were not handled with the least-significant-difference test, since we wished to reduce the type 2 error at the expense of type 1 error. In a recent study with a similar methodology, but with only one post measurement (reducing the number of pairwise comparisons), the same tendencies of the results were observed when using the conservative Bonferroni correction [36]. Another limitation of this study was that there were no control conditions with sham breathing or stimulation; hence, a potential placebo effect cannot be ruled out.

### 4.2. Implications and Future Perspectives

The results of the current study show that the vagus nerve can be stimulated to a similar degree by using DB compared to the effect when using taVNS based on the HRV measures. This could be important for the use of vagus nerve stimulation as a treatment option, since DB is a free alternative that is available to everyone. However, the clinical effects of DB and taVNS in RA and SLE patients should be investigated by using clinical measures, such as disease activity scores, instead of surrogate measures such as HRV. Such studies could be designed as randomized controlled trials in which DB or taVNS is given as an adjunct therapy. Patients could potentially respond better to medications if their HRV would be elevated through VNS, since it has been reported that RA patients with higher HRV respond better to medical treatment [56], but this needs to be tested in future studies. In addition, adherence to DB exercises or taVNS should be investigated to improve the likelihood of patients doing the VNS. The dose–response relationship in patient populations should be investigated to know what doses of VNS are effective when using clinical measures as the outcome and how often the VNS should be performed to maximize the effect. Lastly, the exact physiological mechanisms associated with VNS and, especially, DB should be investigated in more detail.

## 5. Conclusions

DB and taVNS increase HRV parameters significantly in patients with RA and SLE, indicating an increase in vagal tone. This approach could be a potential new treatment option for RA and SLE and, potentially, other autoimmune diseases. However, the clinical effects of these interventions need to be examined more deeply. An increase in HRV parameters was also present in healthy participants after DB and, to a lesser extent, after taVNS, indicating that DB may cause the greatest increase in vagal tone in healthy participants.

## Figures and Tables

**Table 1:** Data are provided as the mean ± standard deviation. For gender, the absolute values and ratio in percent are reported. RA: Rheumatoid arthritis. SLE: Systemic lupus erythematosus. BMI: Body mass index. BP: Blood pressure. DAS-28-CRP: Disease Activity Score-28 C-reactive protein. SLEDAI: Systemic Lupus Erythematosus Disease Activity Index. CDAI: Clinical Disease Activity Index. SLAQ: Systemic Lupus Activity Questionnaire. MDHAQ: Multi-Dimensional Health Assessment Questionnaire. csDMARD: Conventional synthetic disease-modifying anti-rheumatic drugs. bDMARD: Biological disease-modifying anti-rheumatic drugs.

Demographic Data	Healthy Participants (n = 42)	Patients with RA or SLE (n = 52)
Age (years)	28 ± 9	57 ± 13
Sex	Male = 23 (55%)	Male = 12 (23%)
	Female = 19 (45%)	Female = 40 (77 %)
BMI (kg/m^2^)	23.1 ± 5.1	26.2 ± 4.0
BP (mmHG)		
Systolic	123 ± 13	130 ± 19
Diastolic	76 ± 8	81 ± 10
**Patient Disease Characteristics**	**RA (n = 47)**	**SLE (n = 5)**
DAS28-CRP (RA) or SLEDAI (SLE)	2.5 ± 1.1	4 ± 4
CDAI (RA) or SLAQ (SLE)	6.5 ± 7.6	10.0 ± 11.3
MDHAQ	0.5 ± 0.8	0.2 ± 0.4
CRP (mg/L)	5.8 ± 10.4	2.7 ± 2.0
Years since diagnosis	13 ± 10	7 ± 7
Treatment		
csDMARD	n = 38	n = 5
Prednisolone	n = 0	n = 1
bDMARD	n = 28	n = 1

**Table 2:** *p*-values (*p*) and test statistics from the one-way repeated-measure ANOVA tests in the healthy participant population. Post1, Post2, and Post3 were obtained 0–5, 12.5–17.5, and 25–30 min after the interventions, respectively. DB: Deep breathing. taVNS: Transcutaneous auricular vagus nerve stimulation.“B_PX” refers to the comparison between baseline and Post1, Post2, or Post3. “PX_PX” refers to the comparisons between Post1, Post2, and Post3. ↑ shows an increase with respect to the baseline. ↓ shows a decrease with respect to the baseline. Test statistics: F(df (Time), df (Error (Time))) = F-value, *p*-value.

	Test Statistics	B_P1	B_P2	B_P3	P1_P2	P1_P3	P2_P3
SDNN							
taVNS	F(3,123) = 4.11, *p* = 0.008 *	*p* = 0.010 *↑	*p* = 0.822	*p* = 0.054	*p* = 0.006 *↓	*p* = 0.526	*p* = 0.026 *↑
DB	F(3,123) = 11.01, *p* < 0.001 *	*p* < 0.001 *↑	*p* < 0.001 *↑	*p* = 0.005 *↑	*p* = 0.054	*p* = 0.013 *↓	*p* = 0.298
RMSSD							
taVNS	F(3,123) = 2.00, *p* = 0.121	*p* = 0.435	*p* = 0.571	*p* = 0.039 ^#^↑	*p* = 0.908	*p* = 0.091	*p* = 0.072
DB	F(2.5,103.6) = 3.04, *p* = 0.040 *	*p* = 0.158	*p* = 0.009 *↑	*p* = 0.023 *↑	*p* = 0.308	*p* = 0.515	*p* = 0.472
PNN50							
taVNS	F(3,123) = 2.19, *p* = 0.093	*p* = 0.156	*p* = 0.493	*p* = 0.033 ^#^↑	*p* = 0.588	*p* = 0.205	*p* = 0.083
DB	F(3,123) = 3.18, *p* = 0.026 *	*p* = 0.827	*p* = 0.014 *↑	*p* = 0.078	*p* = 0.024 *↑	*p* = 0.113	*p* = 0.290

* marks significant differences (*p* < 0.05). ^#^ indicates *p*-values that were significant in the post hoc test, where rmANOVA found no significant differences.

**Table 3:** *p*-values (*p*) and test statistics from the one-way repeated-measure ANOVA tests in the patient population. Post1, Post2, and Post3 were obtained 0–5, 12.5–17.5, and 25–30 min after the interventions, respectively. DB: Deep breathing. taVNS: Transcutaneous auricular vagus nerve stimulation.“B_PX” refers to the comparison between the baseline and Post1, Post2, or Post3. “PX_PX” refers to the comparisons between Post1, Post2, and Post3. ↑ shows an increase with respect to the baseline. Tests statistics: F(df (Time), df (Error (Time))) = F-value, *p*-value.

	Test Statistics	B_P1	B_P2	B_P3	P1_P2	P1_P3	P2_P3
SDNN							
taVNS	F(3,153) = 19.15, *p* < 0.001 *	*p* < 0.001 *↑	*p* < 0.001 *↑	*p* < 0.001 *↑	*p* = 0.473	*p* = 0.295	*p* = 0.692
DB	F(2.7,136.5) = 15.71, *p* < 0.001 *	*p* < 0.001 *↑	*p* < 0.001 *↑	*p* < 0.001 *↑	*p* = 0.853	*p* = 0.264	*p* = 0.196
RMSSD							
taVNS	F(2.3,118.6) = 8.22, *p* < 0.001 *	*p* = 0.008 *↑	*p* = 0.008 *↑	*p* < 0.001 *↑	*p* = 0.632	*p* = 0.023 *↑	*p* = 0.024 *↑
DB	F(3,153) = 6.42, *p* < 0.001 *	*p* = 0.018 *↑	*p* < 0.001 *↑	*p* < 0.001 *↑	*p* = 0.283	*p* = 0.328	*p* = 0.892
PNN50							
taVNS	F(2.4,122.6) = 7.40, *p* < 0.001 *	*p* = 0.037 *↑	*p* = 0.012 *↑	*p* < 0.001 *↑	*p* = 0.373	*p* = 0.012 *↑	*p* = 0.030 *↑
DB	F(3,153) = 6.53, *p* < 0.001 *	*p* = 0.009 *↑	*p* < 0.001 *↑	*p* < 0.001 *↑	*p* = 0.183	*p* = 0.442	*p* = 0.572

* marks significant differences (*p* < 0.05).

## Data Availability

The data presented in this study are available on request from the corresponding author.
